# Supplementary material for: UBQLN2 is necessary for UBE3A-mediated proteasomal degradation of the domesticated retroelement PEG10
Source: J Cell Sci. 2025 Dec 23;138(24):jcs264105. doi: 10.1242/jcs.264105 (PMC12772957; doi:10.1242/jcs.264105)
Supplement: Supplementary information [file joces-138-264105-s1.pdf]

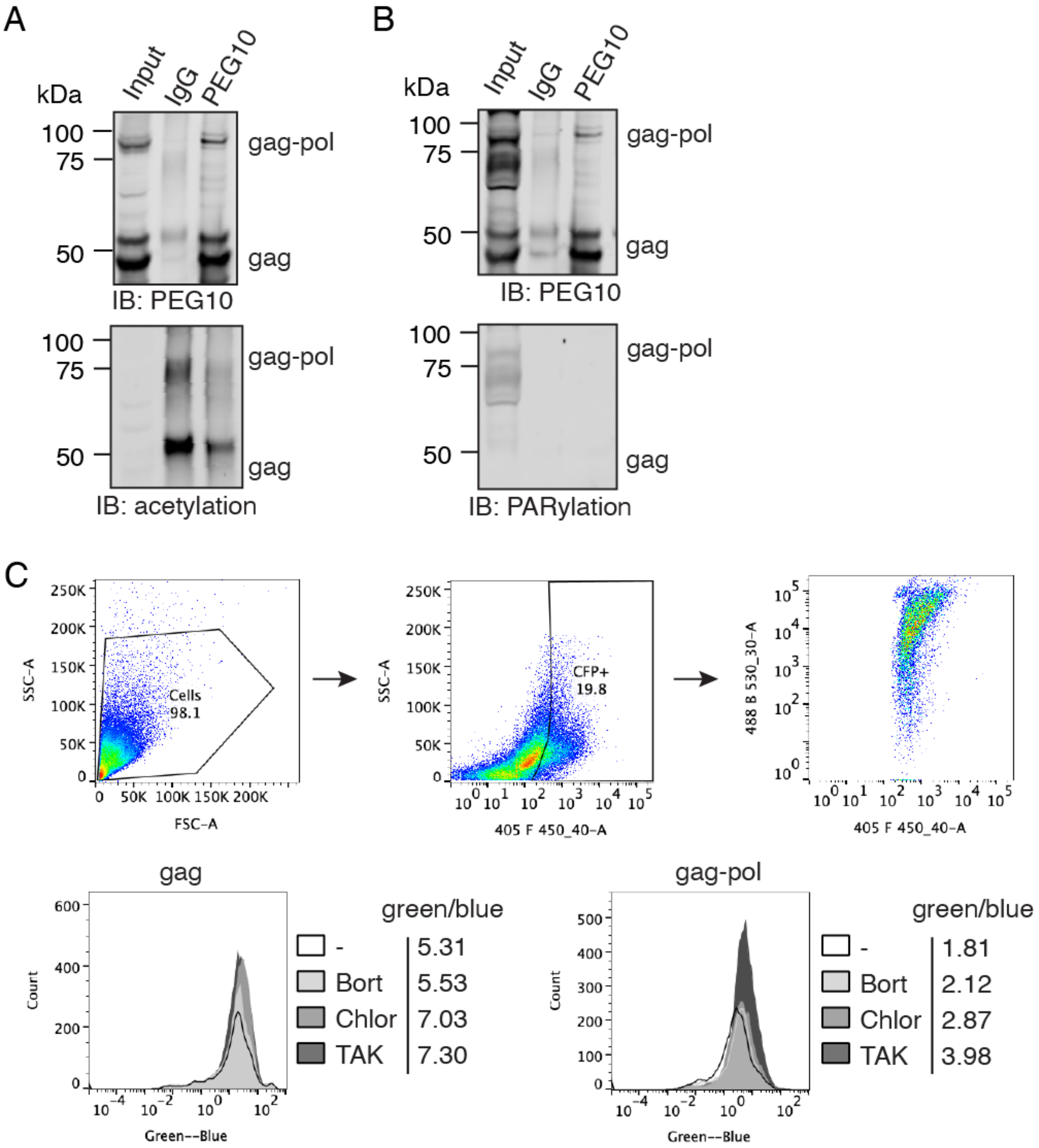


**Fig. S1. Ubiquitination appears to be the dominant modification of PEG10 lysines. A)** Probing PEG10 immunoprecipitation for acetylated lysines. Endogenous PEG10 was precipitated from WT HEK293 lysate and probed for acetylation using antibodies. **B)** Probing PEG10 immunoprecipitation for ADP-ribosylation. Endogenous PEG10 was precipitated from WT HEK293 lysate and probed for ADP-ribosylated lysines **C)** Example dot plot from flow cytometric evaluation of PEG10 abundance. At least 20,000 events are collected for each sample; from this, cells are gated: first as cells (FSC vs. SSC, shown in left), then as CFP+ transfectants (408 vs. SSC, shown on right). Transfected cells are both CFP+ and Dendra2+ (shown on bottom). Then, a per-cell parameter of Dendra2/CFP is generated and Mean Fluorescence Intensity quantified. At bottom are representative histograms for WT HEK293 cell gag and gag-pol transfected wells with Dendra2/CFP parameter shown on x-axis and geometric mean summarized at right.

Journal of Cell Science • Supplementary information

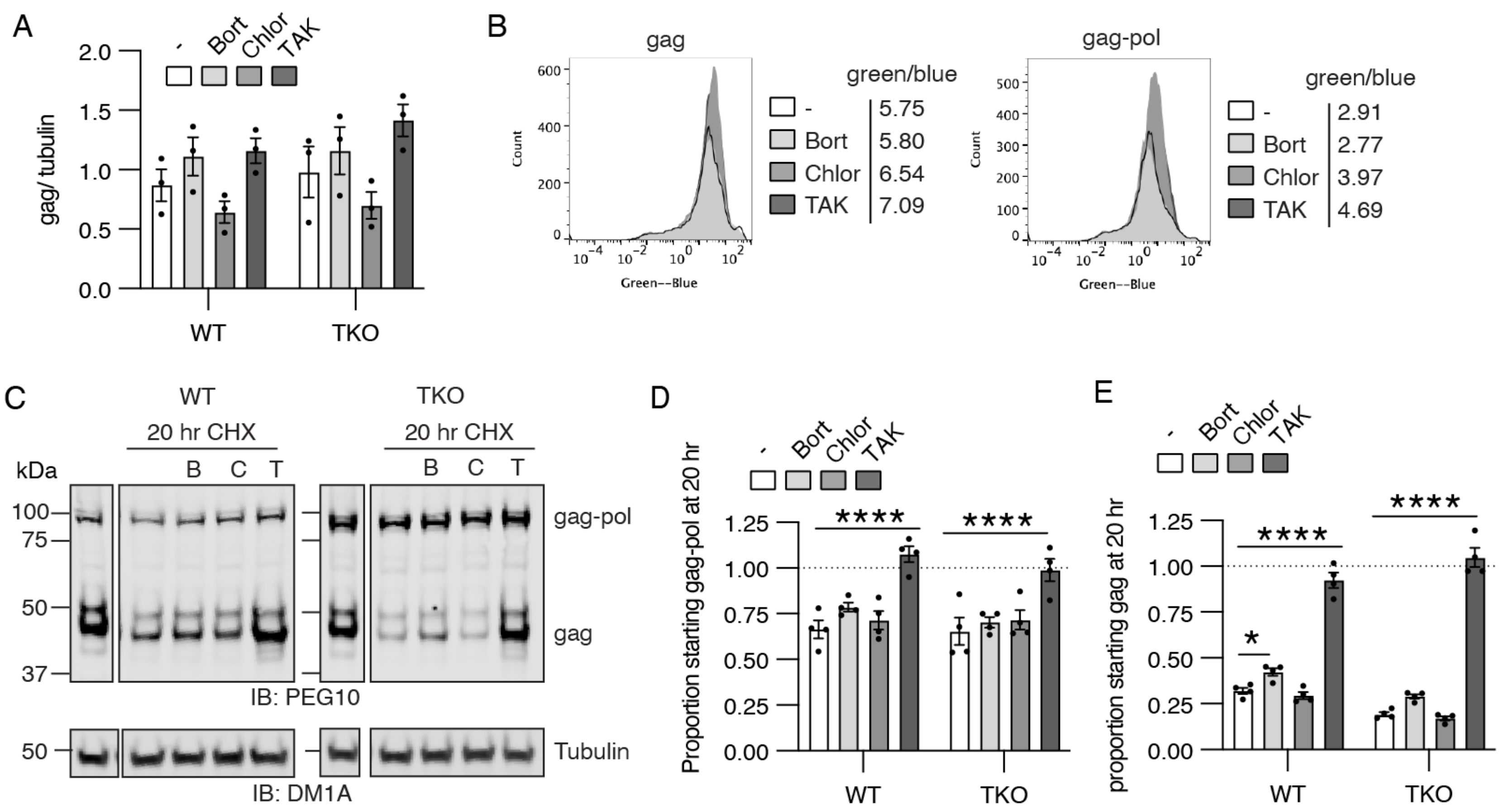


**Fig. S2. Ubiquilin deficient cells accumulate gag-pol protein. A)** Western blot quantification of gag protein from Fig. 2C. There are no statistically significant differences in gag expression upon any drug treatment as measured by two-way ANOVA with multiple comparisons. **B)** Representative histograms for TKO HEK293 cell gag and gag-pol transfected wells with Dendra2/CFP parameter shown on x-axis and geometric mean summarized at right. **C)** Cycloheximide treatment over 20 hours in the presence of bortezomib (“B”), chloroquine(“C”), or TAK243 (“T”). After 20 hours with drug(s), cells were harvested and lysed for PEG10 western blot. Shown is one of four representative experiments. **D)** Quantification of data from (C). The proportion of starting gag-pol (leftmost column for each cell type in (C) and set to 1.0 for each replicate and cell line) was quantified for each experiment. The difference between cycloheximide-treated and cycloheximide- and TAK243-treated cells was significant for each cell line as measured by ANOVA with multiple comparisons; all other comparisons were not significant. n=4 independent experiments. **E)** Cycloheximide chase results for gag protein from (C). Statistics were determined by two-way ANOVA with multiple comparisons; n=4 independent experiments. For (D-E), shown is mean ± SEM.

Journal of Cell Science • Supplementary information

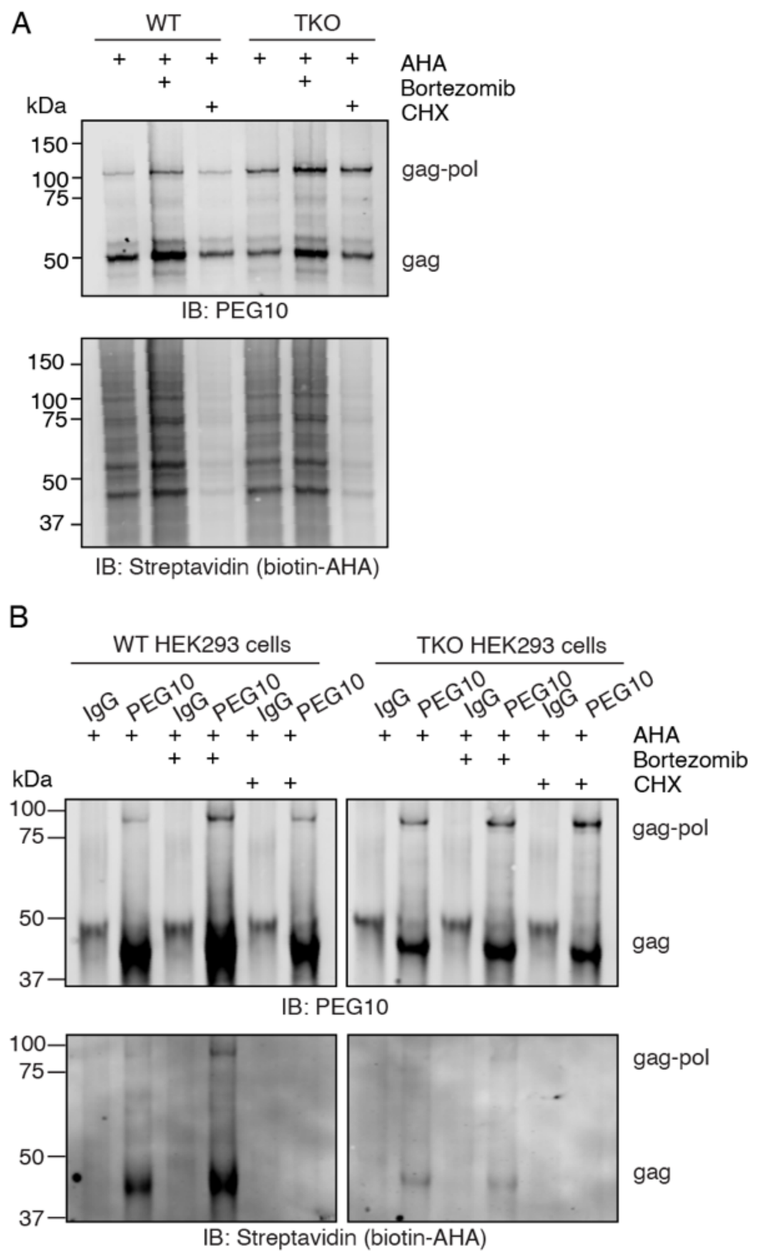


**Fig. S3. Protein synthesis is not affected by Ubiquilin knockouts. A)** Western blot of whole cell lysate following incubation of live cells for four hours with methionine analog azido-homo-alanine, followed by biotin-alkyne addition. Some samples were co-treated with Bortezomib or cycloheximide as a control. Lysate was probed against PEG10 with polyclonal antibody (top) or AHA incorporation with streptavidin (bottom). **B)** Western blot of metabolically labeled PEG10 protein from 4 hours of protein synthesis in WT and TKO cells. Some samples were co-treated with bortezomib or cycloheximide as a control. PEG10 was immunoprecipitated from cells with polyclonal PEG10 antibody, then blotted against PEG10 and biotin-AHA with streptavidin. Shown is one of two representative experiments.

Journal of Cell Science • Supplementary information

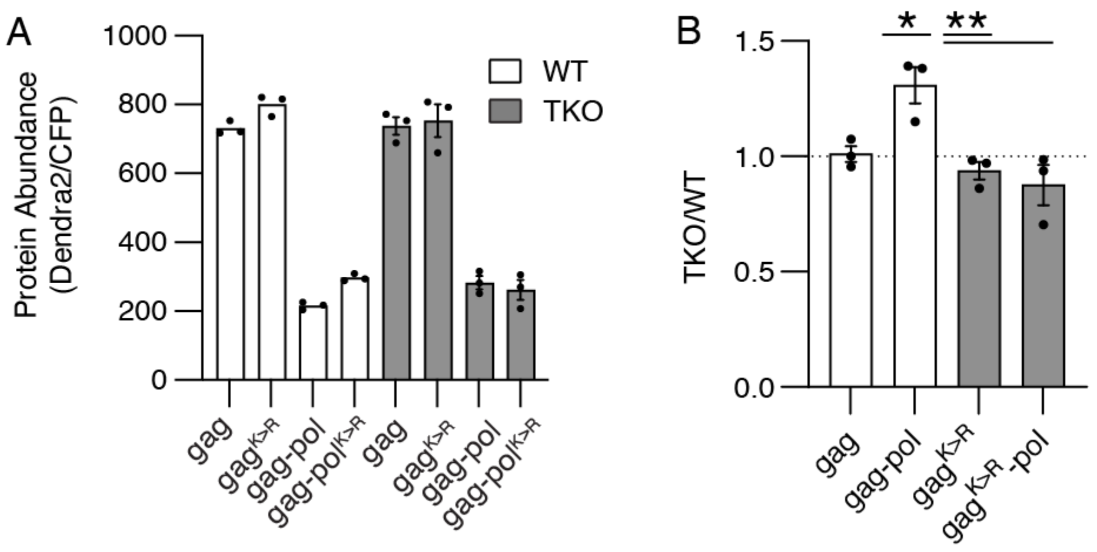


**Fig. S4. Mutation of gag lysine residues impacts protein abundance and dependence on Ubiquilins. A)** A gag construct with all lysines mutated to arginine was cloned and used to test the importance of gag lysine residues to protein degradation. The client accumulation assay was performed in WT (left), and TKO (right) HEK293 cells. **B)** TKO/WT values are shown. For (A-B), n=3 and statistics were determined via one-way ANOVA and multiple comparisons test. Shown is mean ± SEM.

Journal of Cell Science • Supplementary information

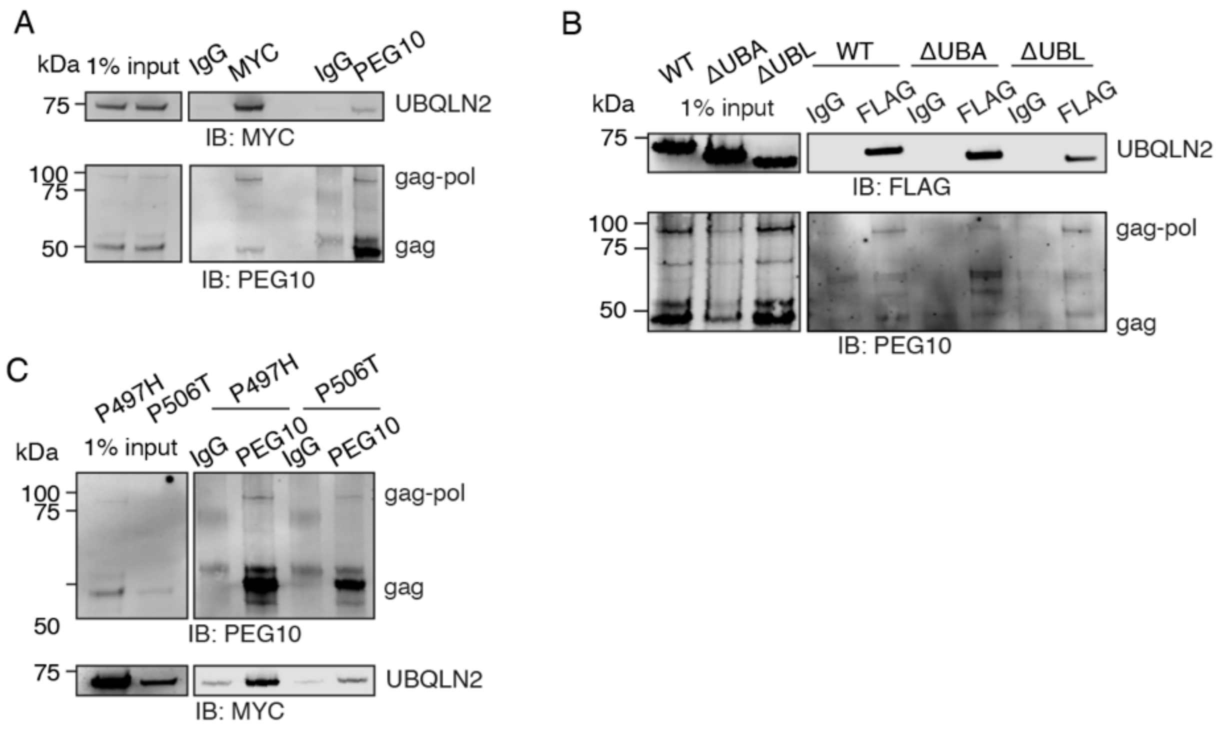


**Fig. S5. Binding of UBQLN2 to PEG10. A)** Reciprocal immunoprecipitation of MYC-UBQLN2 (left) or endogenous PEG10 (right) shown on the same blot. MYC-UBQLN2-expressing TKO cells were lysed for immunoprecipitation. PEG10 was detected with polyclonal antibody. Shown is one of two representative experiments. **B)** TKO HEK293 cells were transiently transfected with N-term 3xFLAG-UBQLN2 constructs and lysate was subjected to an immunoprecipitation using an anti-FLAG antibody or IgG control antibody followed by western blot. PEG10 was detected with polyclonal antibody, and UBQLN2 was detected with FLAG antibody. n=1. **C)** ALS-linked mutant MYC-UBQLN2-expressing TKO HEK293 cell lysate was subjected to an immunoprecipitation of endogenous PEG10, followed by western blot analysis. UBQLN2 mutants were detected by an N-term MYC-tag. Shown is one of two representative experiments.

Journal of Cell Science • Supplementary information

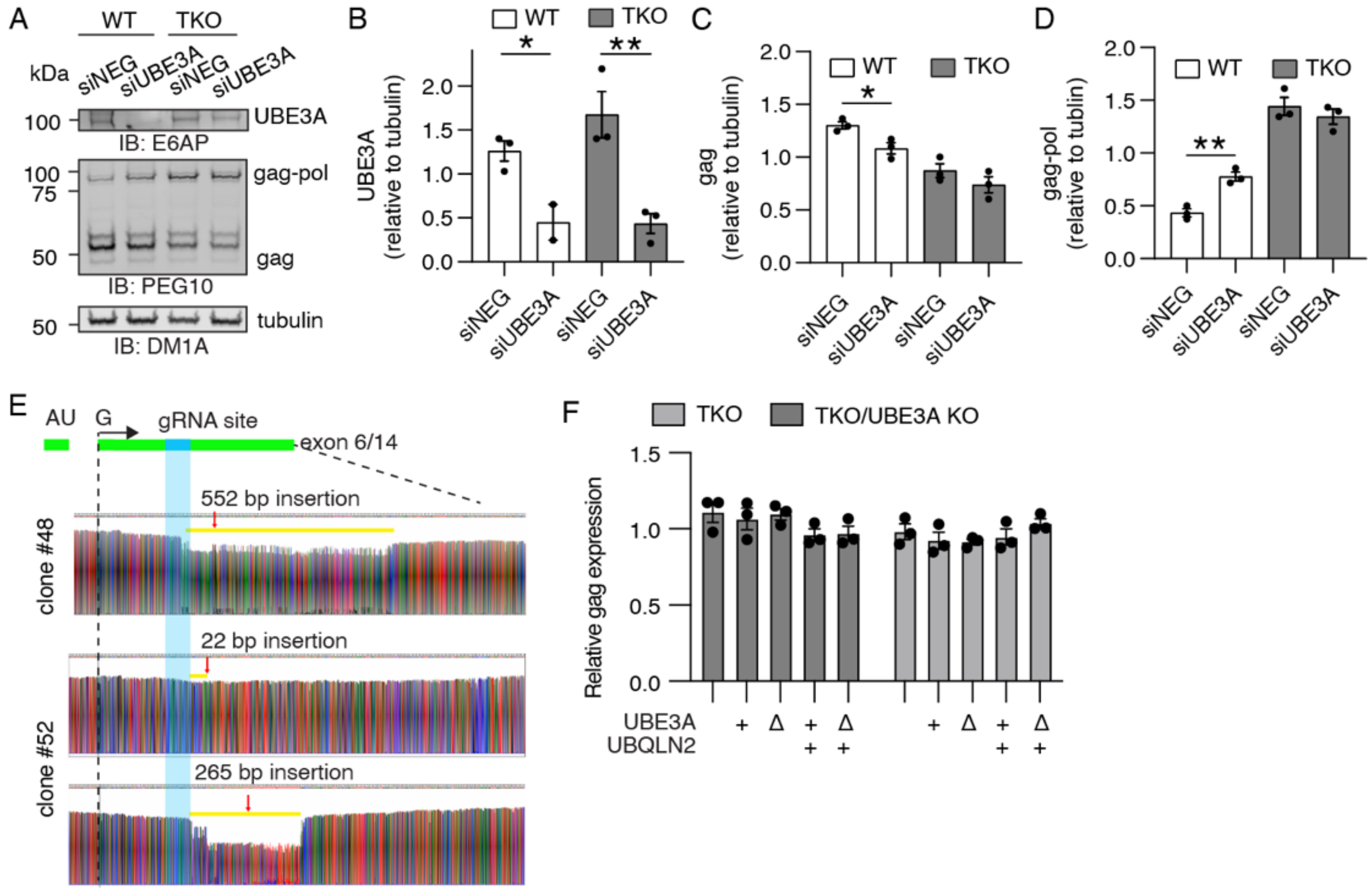


**Fig. S6. Single targeting siRNA against *UBE3A* also leads to PEG10 gag-pol accumulation only when UBQLN2 is present. A)** Representative blot showing single siRNA knockdown of UBE3A. Shown is one of three representative blots. **(B-D)** Quantification of western blot analysis from single si*UBE3A* siRNA in WT and TKO HEK293 cells. UBE3A (B), gag (C), and gag-pol (D) were quantitated. For (B-D), samples are normalized to a blot average to account for variance in western blot detection. Shown is mean ± SEM for three independent experiments. Statistics were determined using two-way ANOVA and multiple comparisons test. **E)** Long-read sequencing from PCR amplified regions of gDNA from CRISPR-mutated UBE3A clones. At the top is a schematized view of a small region of the UBE3A gene, with the tail end of exon 5 and exon 6 out of 14 exons of the UBE3A gene shown in green. Note the translational start site is generated through removal of the intron between exons 5 and 6. Selected gRNA site is shown in blue in the approximate middle of exon 6. Two clones were selected because of the absence of UBE3A band by western blot: clone #48 and clone #52. DNA insertions immediately flanking the gRNA site are highlighted in yellow, and the most proximal stop codon is shown with a red arrow. **F)** Quantification of gag from UBE3A rescue experiment in Fig. 6F-G. gag levels were quantified, normalized to Tubulin of each well, and then normalized to a blot average. Shown is mean ± SEM for three independent experiments. Statistics were determined by two-way ANOVA with multiple comparisons.

Journal of Cell Science • Supplementary information

Fig. 1

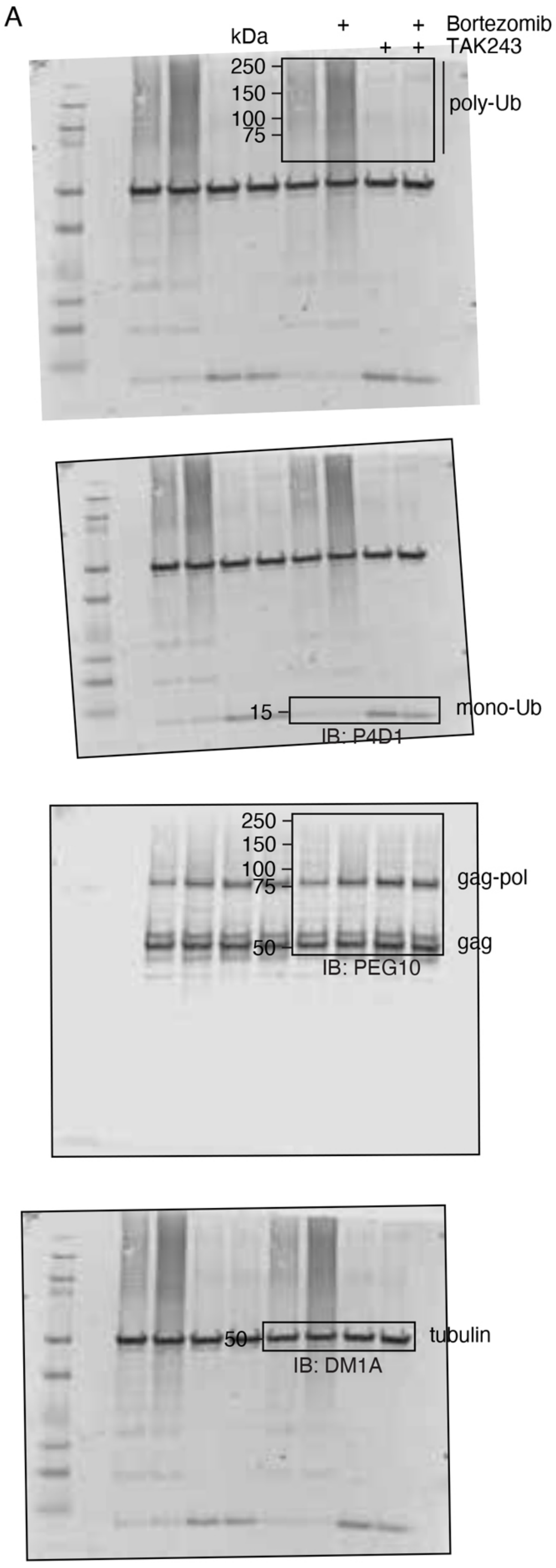


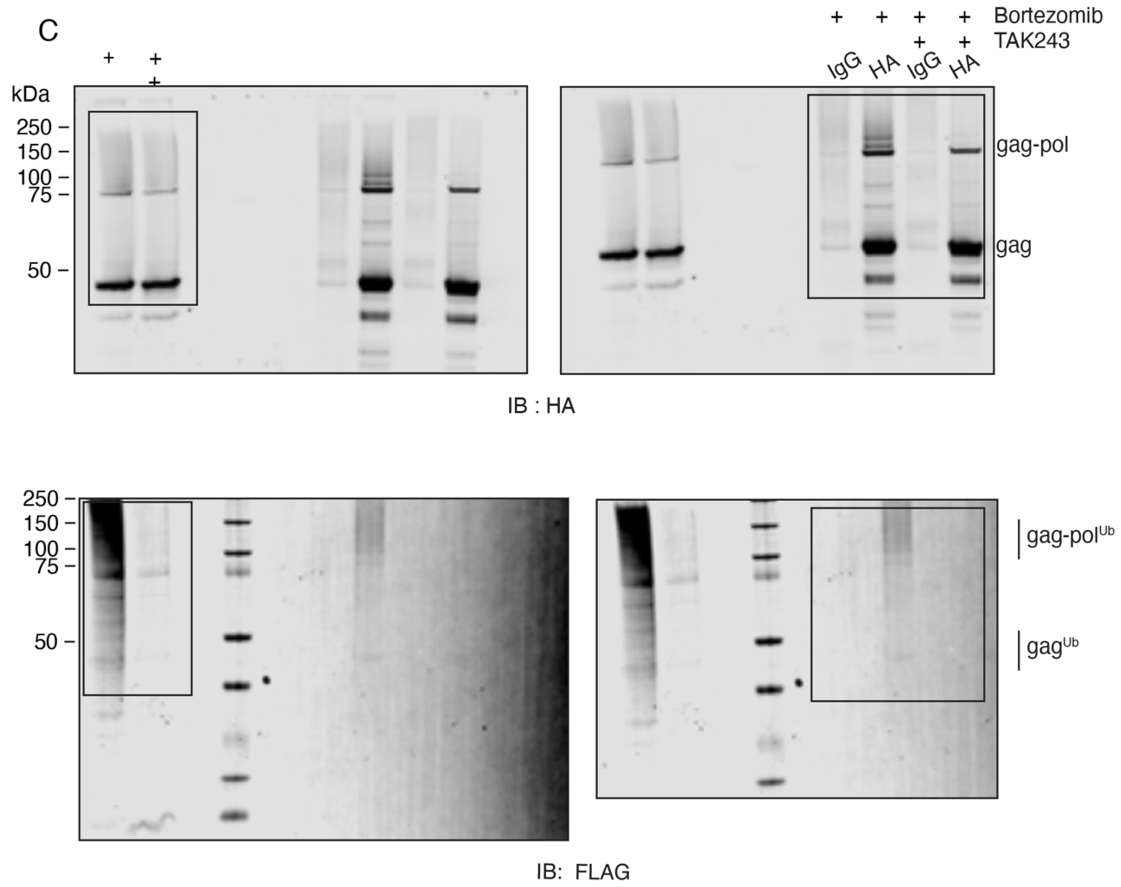


Journal of Cell Science • Supplementary information

Fig. 2

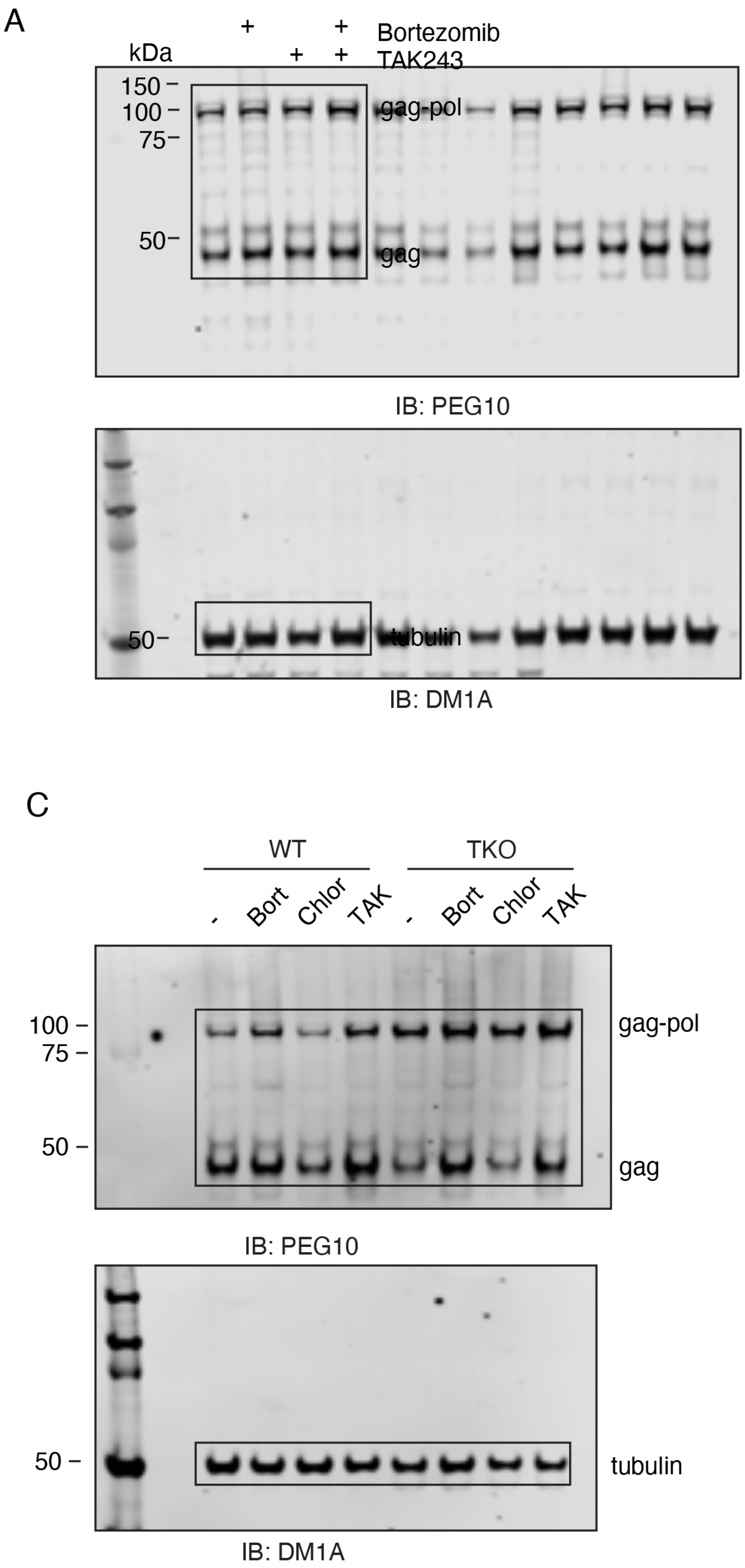


Journal of Cell Science • Supplementary information

Fig. 4

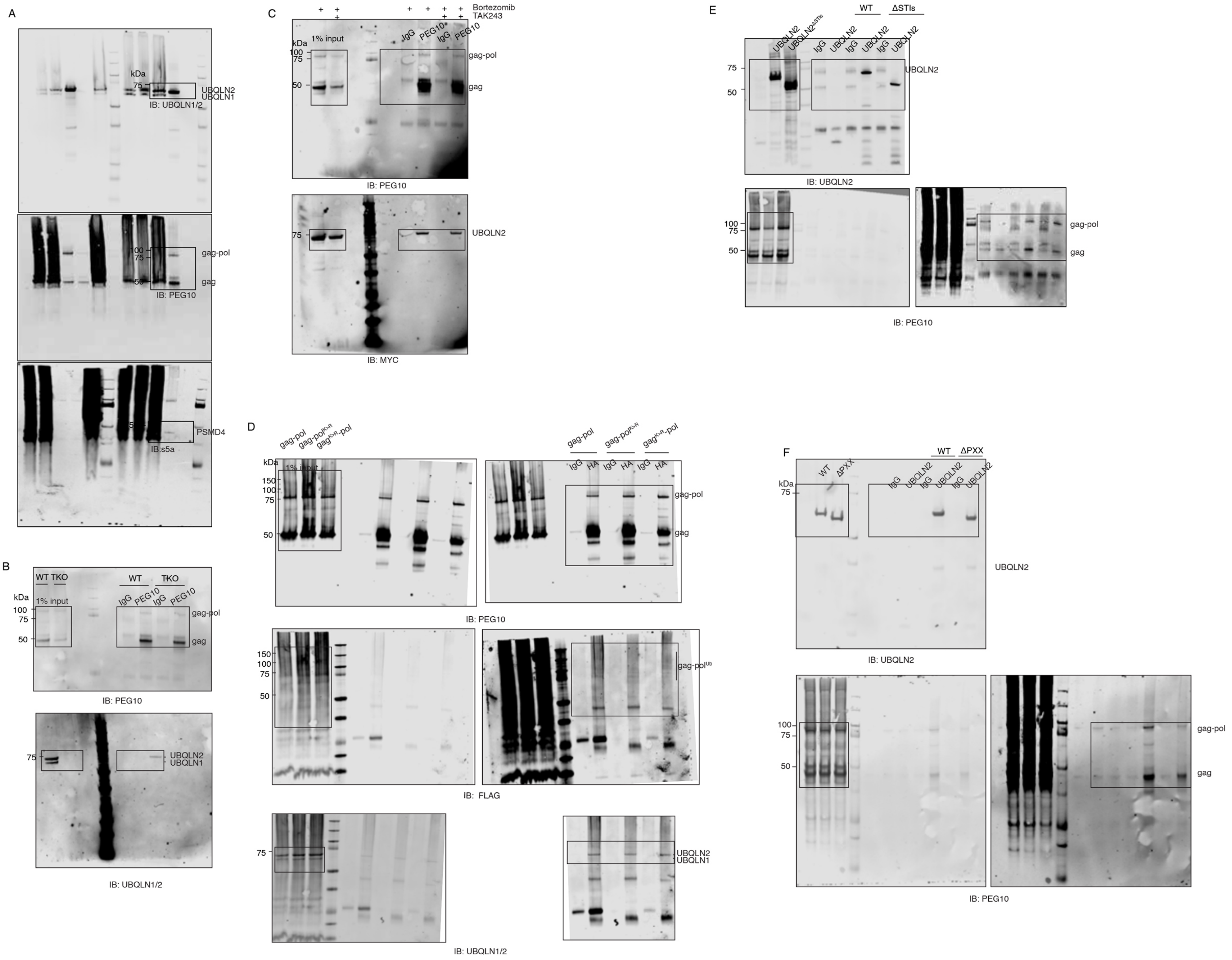


Journal of Cell Science • Supplementary information

Fig. 5

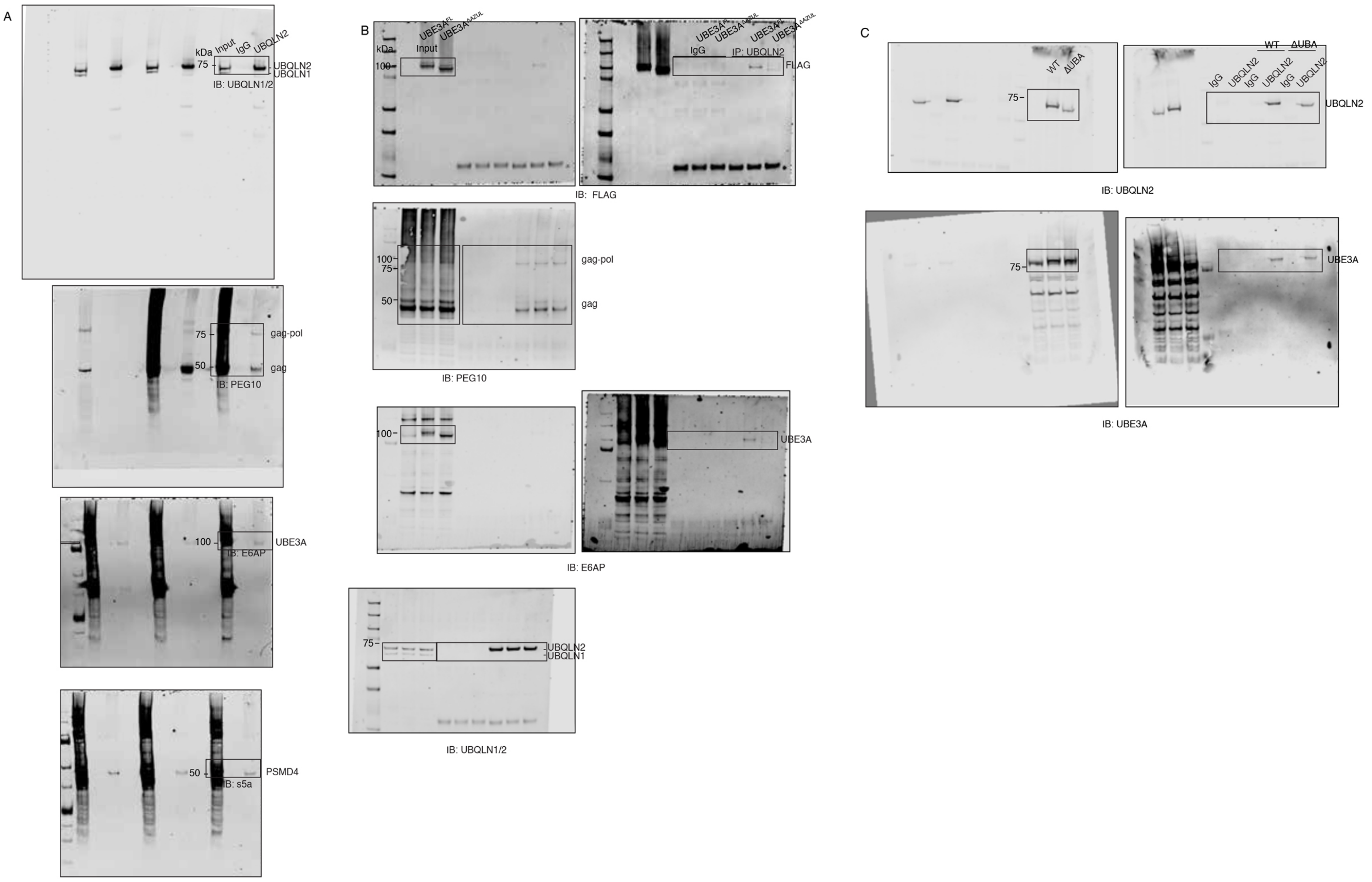
A
kDa
Input
IgG
UBQLN2
75
UBQLN2
UBQLN1
IB: UBQLN1/2
75
50
gag-pol
gag
IB: PEG10
100
UBE3A
IB: E6AP
50
IB: s5a
PSMD4
B
kDa
Input
100
IgG
IP: UBQLN2
FLAG
IB: FLAG
100
75
50
gag-pol
gag
IB: PEG10
100
UBE3A
IB: E6AP
75
UBQLN2
UBQLN1
IB: UBQLN1/2
C
WT
ΔUBA
75
IgG
UBQLN2
IgG
UBQLN2
UBQLN2
IB: UBQLN2
75
UBE3A
IB: UBE3A

Fig. 6

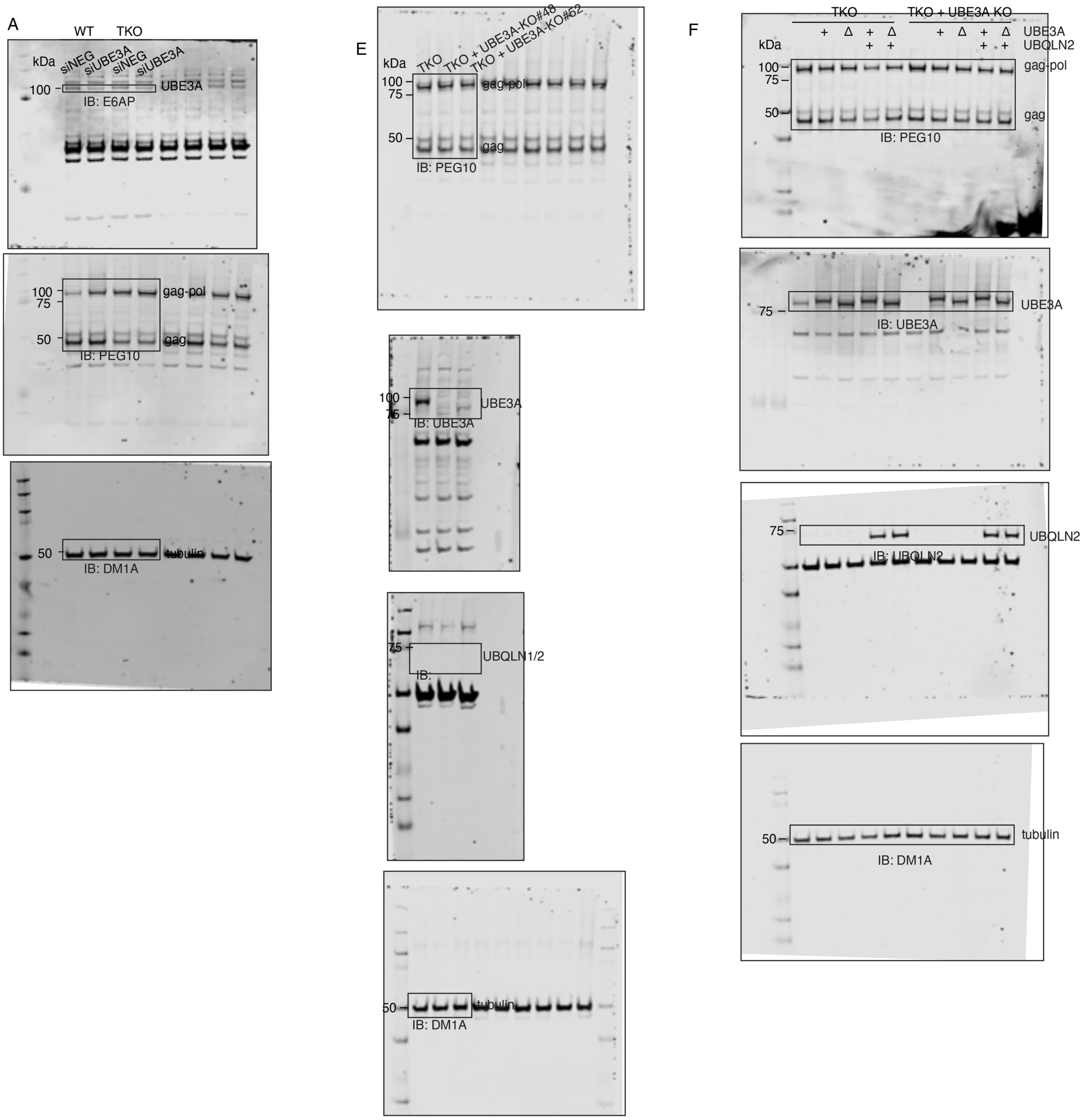


**Fig. S7. Blot Transparency**

Journal of Cell Science • Supplementary information
